# NVP-BEZ235, a dual PI3K/mTOR inhibitor synergistically potentiates the antitumor effects of cisplatin in bladder cancer cells

**DOI:** 10.3892/ijo.2014.2505

**Published:** 2014-06-19

**Authors:** DU G. MOON, SANG E. LEE, MI M. OH, SANG C. LEE, SEONG J. JEONG, SUNG K. HONG, CHEOL Y. YOON, SEOK S. BYUN, HONG S. PARK, JUN CHEON

**Affiliations:** 1 Department of Urology, College of Medicine, Korea University, Guro Hospital, Seoul, Republic of Korea; 2 Korea University Center of Regenerative Medicine, Seoul, Republic of Korea; 3 Department of Urology, School of Medicine, Seoul National University, Bundang Hospital, Gyeonggi-do, Republic of Korea; 4 Department of Urology, College of Medicine, Korea University, Anam Hospital, Seoul, Republic of Korea

**Keywords:** urinary bladder, carcinoma, resistance, cisplatin, NVP- BEZ235

## Abstract

The PI3K/Akt/mTOR pathway is a prototypic survival pathway and constitutively activated in many malignant conditions. Moreover, activation of the PI3K/Akt/mTOR pathway confers resistance to various cancer therapies and is often associated with a poor prognosis. In this study, we explored the antitumor effect of NVP-BEZ235, a dual PI3K/mTOR inhibitor in cisplatin-resistant human bladder cancer cells and its synergistic interaction with cisplatin. A human bladder cancer cell line with cisplatin resistance was exposed to escalating doses of NVP-BEZ235 alone or in combination with cisplatin and antitumor effects was determined by the CCK-8 assay. Based on a dose-response study, synergistic interaction between NVP-BEZ235 and cisplatin was evaluated by combination index (CI), three-dimensional model and clonogenic assay. The combination of NVP-BEZ235 and cisplatin caused significant synergistic antitumor effect in cisplatin-resistant bladder cancer cells over a wide dose range and reduced the IC_50_ of NVP-BEZ235 and cisplatin by 5.6- and 3.6-fold, respectively. Three-dimensional synergy analysis resulted in a synergy volume of 388.25 μM/ml^2^% indicating a strong synergistic effect of combination therapy. The combination therapy caused cell cycle arrest and caspase-dependent apoptosis. Although NVP-BEZ235 suppressed PI3K/mTOR signaling without any paradoxical induction of Akt activity, it caused MEK/ERK pathway activation. The present study demonstrated that the PI3K/mTOR dual inhibitor NVP-BEZ235 can synergistically potentiate the antitumor effects of cisplatin in cisplatin-resistant bladder cancer cells though the suppression of cell cycle progression and the survival pathway as well as induction of caspase-dependent apoptosis.

## Introduction

Cisplatin-based combination chemotherapy (i.e., M-VAC or G/C) in a mainstay treatment for metastatic bladder cancer showing a 50–70% response rate and a 15–20% improvement in survival. However, most patients have a recurrence within the first year and in the recurrent cases, additional cisplatin combination chemotherapy provides limited benefit (1,2). Thus, over the past three decades there have been numerous trials to overcome cisplatin resistance in bladder cancer.

Advances in the understanding of tumor biology have established the critical role of targeted therapy as first- or second-line treatment options for various malignant diseases including genitourinary tumors. In addition, a growing body of data suggests that the combination of targeted agents is a promising strategy to enhance the antitumor effect of conventional chemotherapies (3–5). PI3K (phosphatidylinositol 3-kinase)/Akt (protein kinase B)/mTOR (mammalian target of rapamycin) signaling axis is a major survival pathway and its abnormal activation is frequently involved in the development and progression of various tumors including invasive bladder cancers (6–9). Moreover, several studies have observed a synergistic antitumor effect between PI3K or mTOR inhibitors and conventional chemotherapy agents such as cisplatin in chemo-naïve or resistant cancers like melanoma, ovarian, and nasopharyngeal cancer (10–12). These findings provide a rationale for targeting PI3K/Akt/mTOR survival pathway for the treatment of patients with advanced bladder cancer, especially after failure of first-line chemotherapy such as cisplatin-based regimens. Based on these ideas, a few studies have assessed and demonstrated the antitumor effect of mTOR inhibitors for bladder cancers in pre-clinical or clinical settings (13–15). However, no study has tested in detail the synergistic effect between cisplatin and mTOR inhibitors in human bladder cancers, especially for cisplatin-resistant tumors (16,17).

We report that the PI3K/mTOR dual inhibitor NVP- BEZ235, an orally bioavailable imidazoquinoline derivative synergistically potentiates the antitumor effect of cisplatin in bladder cancer cells.

## Materials and methods

### Cell lines and chemicals

Bladder cancer cell lines (J82, SW1710, T24, HTB5, HTB9, UMUC14 and 253J) were maintained in MEM, DMEM, and RPMI-1640 supplemented with 10% fetal bovine serum (Mediatech, Herndon, VA, USA) and 100 U/ml penicillin/100 mg/l streptomycin (Gibco BRL, Grand Island, NY, USA) with 5% CO_2_ at 37°C. UMUC14 was donated by Professor E.S. Lee (Seoul National University, Seoul, Korea) and all other cells lines obtained from ATCC (American Type Culture Collection, Manassas, VA, USA) or KCLB (Korean Cell Line Bank, Seoul, Korea). The cisplatin-resistant T24R2 cell line was established by serial desensitization of T24 cells. NVP-BEZ235 was kindly provided by Norvatis Pharmaceuticals Inc. (Basel, Switzerland). Everolimus and temsirolimus were purchased from LC laboratories (Woburn, MA, USA). Cisplatin was obtained from Pfizer (Pfizer Korea Ltd., Seoul, Korea). NVP-BEZ235 compound was dissolved in 100% DMSO at 85°C to prepare 10 mM stock solution and kept at 4°C before use.

### Cytotoxicity assay

Cells were treated with cisplatin (0.039–40.0 μg/ml), NVP-BEZ235 (0.19 nM-20.0 μM), temsirolimus (0.313–10.0 μM) or, everolimus (0.313–10.0 μM) and the antitumor effect was determined by CCK-8 assay (Cell counting kit-8; Dojindo Molecular Technologies, Gaithersburg, MD, USA) according to the manufacturer’s instructions.

### Determination of synergism

The synergy between drugs was determined by combination index (CI) in which CI values <1.0, >1.0, and 1.0 indicated synergism, antagonism, and additivity, respectively (18).

Data were also analyzed using the MacSynergy II software program at 95% confidence limits. The degree of interaction is expressed for data represented as percentages in which 0–25, 25–50, 50–100 and >100 μM/ml^2^% calculated values in either a positive (synergy) or negative (antagonism) direction are defined as insignificant, minor, moderate and strong interaction respectively (19–21).

### Clonogenic assay

T24R2 cells (2×10^2^) were plated in a 6-well culture plate and treated with NVP-BEZ235 (0.5 μM) and/or cisplatin (0.5 μg/ml) for 48 h with 5% CO_2_ at 37°C. After washing with PBS, cells were maintained for another 10 days and visualized by 0.4% crystal violet staining.

### Flow cytometric analysis of cell cycle and apoptosis

T24R2 cells were treated with NVP-BEZ235 (0.5 μM) and/or cisplatin (0.5 μg/ml) for 48 h, fixed in 70% ethanol, and stained with a propidium iodide solution [970 μl PBS and 40 μl of 1 mg/ml propidium iodide (Sigma)] and 3 μl of RNase A (Sigma). Alteration in the cell cycle was determined by FACSCalibur flow cytometer (Becton-Dickinson, San Jose, CA, USA).

Induction of apoptosis by combination treatment was also assessed using Annexin V-FITC apoptosis detection kit (BP Pharmingen, San Jose, CA, USA) according to the manufacturer’s instructions. Triplicate study results (≥5,000–10,000 counts per each study) were used for the quantitative analysis.

### Hoechst 33342 nuclear stating

T24R2 cells were exposed to NVP-BEZ235 (0.5 μM) and/or cisplatin (0.5 μg/ml) for 48 h and fixed with 4% paraformaldehyde before staining with 0.5 ml of Hoechst 33342 (10 μg/ml; Sigma-Aldrich, St. Louis, MO, USA) for 30 min at 37°C in the dark.

### Analysis of apoptosis-, cell cycle- and survival-related protein expression

After 48 h of treatment with NVP-BEZ235 (0.5 μM) and/or cisplatin (0.5 μg/ml), protein was extracted from T24R2 cells using RIPA buffer, fractionated by SDS-PAGE, transferred to PVDF membrane, and incubated with the corresponding primary antibodies [cyclin A, cyclin B1, cyclin D1, CDC2C, p-CDC2C (Tyr15), CDC25C, p-CDC25C (Ser216), pRb (Ser807/811), cleaved cleaved caspase (-3, -8 and -9), cleaved PARP, cIAP1, cIAP2, XIAP, survivin, p-IKKα (Ser176/180), IKKα, p-IκBα (ser32), IκBα, NF-κB, p-Akt (ser473), Akt, p-PI3K (Tyr199/458), PI3K, p-mTOR (Ser2448), mTOR, p-p70S6K (Thr389), p70S6K, p-GSK-3β (Ser9), GSK-3β, p-4E-BP1 (Thr37/46), 4E-BP1, p-MEK1/2 (Ser217/221), MEK1/2, p-ERK1/2 (Thr202/Tyr204), and ERK1/2; Cell Signaling Technology, Danvers, MA, USA; Bcl-2, Bax and Bad; Santa Cruz Biotechnology, Santa Cruz, CA, USA] and secondary antibodies before signal detection with an enhanced chemiluminescence Western blot substrate kit (Pierce, Rockford, IL, USA).

### Statistical analysis

All statistical analyses were carried out using the SPSS 14.0K software (SPSS Inc., Chicago, IL, USA). Unless indicated otherwise, the data sets consist of at least three biological replicates, and the data are expressed as the mean ± SD. Statistical significance was determined by a two-sample t-test, and null hypotheses of no difference were rejected if p-values were <0.05.

## Results

### Antitumor effect of NVP-BEZ235 in bladder cancer cells

**Figure 1:**
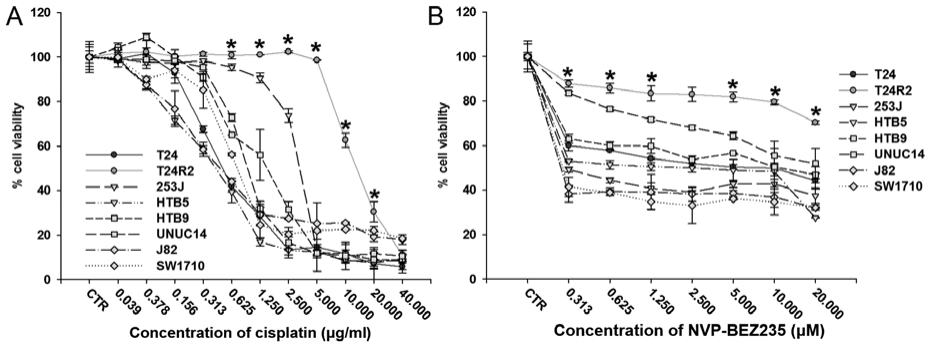
Antitumor effect of cisplatin (A) and NVP-BEZ235 (B) in bladder cancer cells. Human transitional cell carcinoma cell lines (HTB9, grade 2; J82, grade 3; SW1710, grade 3; T24, grade 3; T24R2, grade 3; HTB5, grade 4; UMUC14, grade 4; and 253J, grade 4) were exposed to increasing doses of cisplatin (0.039–40.0 μg/ml) or NVP-BEZ235 (0.313–20 μM) for 72 h and antitumor effect in each cell line was assessed by CCK-8 assay. CTR stands for untreated control and asterisks denote significant difference between T24 and T24R2 (p<0.05). Each data point represents the mean ± SD of triplicate experiments.

After 72 h of exposure, T24R2 showed virtually no response to cisplatin treatment up to 5.0 μg/ml concentration, while the proliferations of all other bladder cancer cell lines tested were nearly completely inhibited by cisplatin in the same dose ranges (Fig. 1A). NVP-BEZ235 exerted a dose-dependent but varying antitumor effect on bladder cancer cells. However, T24R2 required a significantly higher amount of NVP-BEZ235 for the proliferation suppression to a similar level as other bladder cancer cell lines including T24 (Fig. 1B).

**Figure 2:**
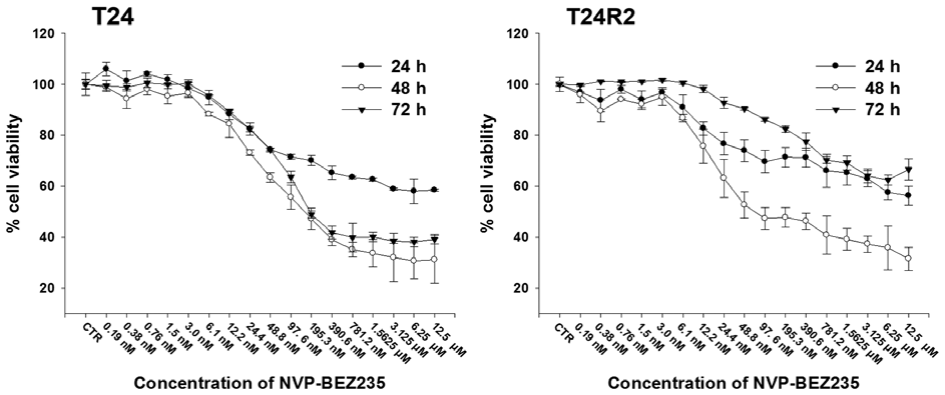
Dose response studies of NVP-BEZ235 in T24 and T24R2 cells. Both cell lines were treated with increasing doses of NVP-BEZ235 (0.19 nM-12.5 μM) for 24–72 h and changes in cell proliferation were examined by CCK-8 assay. Each data point represents the mean ± SD of triplicate experiments.

Up to 48 h, NVP-BEZ235 exerted dose- and time-dependent antitumor effect in both T24 and T24R2 cells. However, when exposed for >72 h, both cells at least partly recovered from NVP-BEZ235-induced suppression and regained their proliferation to the similar level as 24- (T24R2) and 48-h (T24) treatment, respectively (Fig. 2).

**Figure 3:**
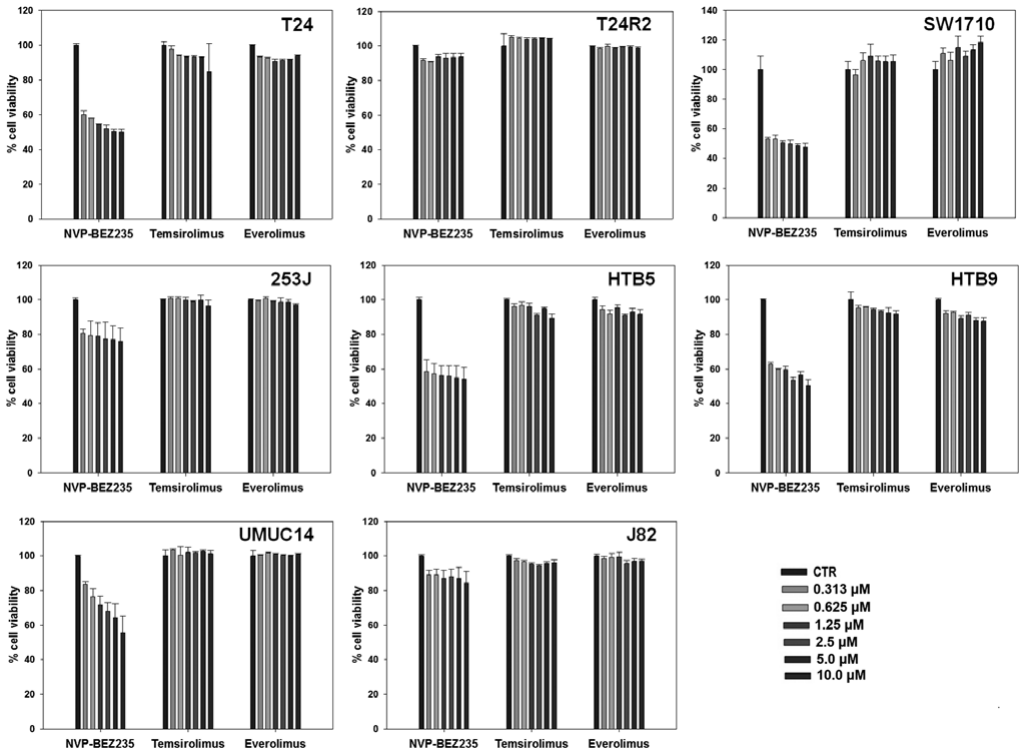
Comparative analysis of antitumor effect of mTOR and PI3K/mTOR dual inhibitor in bladder cancer cells. Bladder cancer cell lines (T24, T24R2, 253J, HTB5, HTB9, UMUC14, J82 and SW1710) were exposed to equimolar concentration (0.313–10.0 μM) of temsirolimus, everolimus, and NVP-BEZ235 for 72 h and the antitumor effect of each drug was determined by CCK-8 assay. Each data point represents the mean ± SD of triplicate experiments.

At equimolar concentrations NVP-BEZ235 exerted relatively more potent antitumor effect compared with mTOR inhibitors (temsirolimus, everolimus) against bladder and breast cancer cells (Fig. 3).

### Synergistic antitumor effect of NVP-BEZ235 and cisplatin in bladder cancer cells

**Figure 4:**
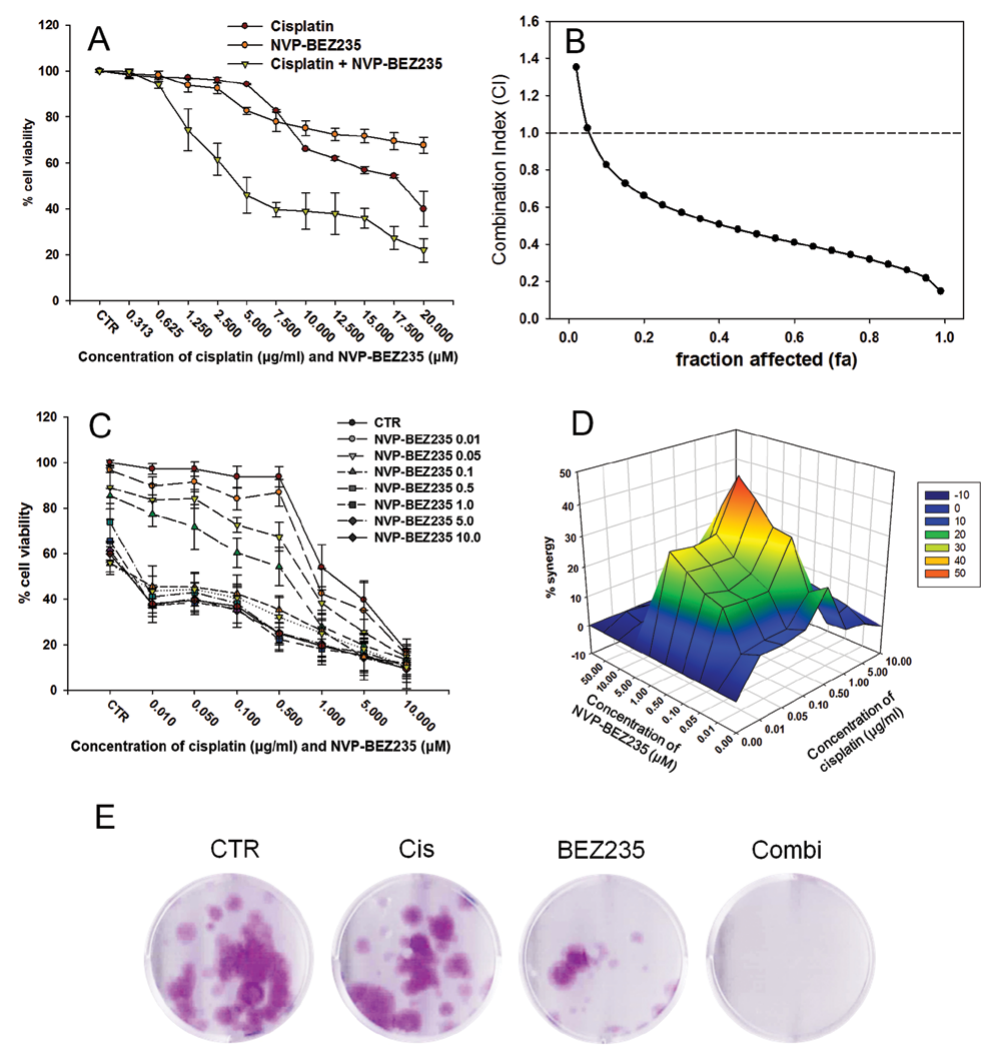
Synergistic antitumor effect between NVP-BEZ235 and cisplatin in cisplatin-resistant bladder cancer cells 72 h after treatment. Dose-response study (A) of 1:1 fixed ratio combination of NVP-BEZ235 (0.313–20 μM) and cisplatin (0.313–20 μg/ml) against T24R2 cells and (B) fa-CI plot in which fa and CI indicate fraction affected, and combination index, respectively. CI<1, CI=1, and CI>1 denote synergistic, additive, and antagonistic interaction, respectively. Each data point represents the mean ± SD of triplicate experiments. For more detailed analysis of synergism, four independent combination experiments of NVP-BEZ235 (0.01–10.0 μM) and cisplatin (0.01–10 μg/ml) were performed and the results were three-dimensionally reconstructed by MacSynergy II data analysis program where peak, depression, and horizontal plane indicate the interactions were synergistic, antagonistic, and additive, respectively (C and D). (E) Clonogenic assay of T24R2 cells exposed to NVP-BEZ235 (0.5 μM) alone or in combination with cisplatin (0.5 μg/ml). CTR, Cis, BEZ235 and Combi stand for untreated control, cisplatin single treatment, NVP-BEZ235 single treatment and combination treatment respectively.

To test synergistic effect T24R2 cells were exposed to increasing doses of NVP-BEZ235 alone or in a 1:1 fixed ratio combination with cisplatin and antitumor effect was assessed by CCK-8 assay (Fig. 4A). Correlation coefficient values (r) ranged from 0.926 to 0.988 indicating data conforming to median effect principle (Table I). The IC_50_s (Dm) of NVP-BEZ235 was 37.47 μM, cisplatin was 23.89 μg/ml, and the combination 6.63 μM (Table I). The isobole and DRI (dose reduction index) analysis showed a CI<1 over a wide range of fractions affected (fa, 0.1–0.99) (Fig. 4B and Table II).

For a more detailed evaluation of synergy, four independent combination experiments of NVP-BEZ235 (0.01–10.0 μM) and cisplatin (0.01–10 μg/ml) were performed to generate synergy plot using MacSynergy II data analysis program (Fig. 4C). There was strong synergy between NVP-BEZ235 and cisplatin in T24R2 cells (synergy volume of 388.25 μM/ml^2^%) with minimal antagonism (-2.41 μM/ml^2^%) (Fig. 4D). Clonogenic assay also showed synergistic interaction between NVP-BEZ235 and cisplatin for T24R2 cells (Fig. 4E).

### NVP-BEZ235 and cisplatin combination induces S phase cell cycle arrest and apoptosis

**Figure 5:**
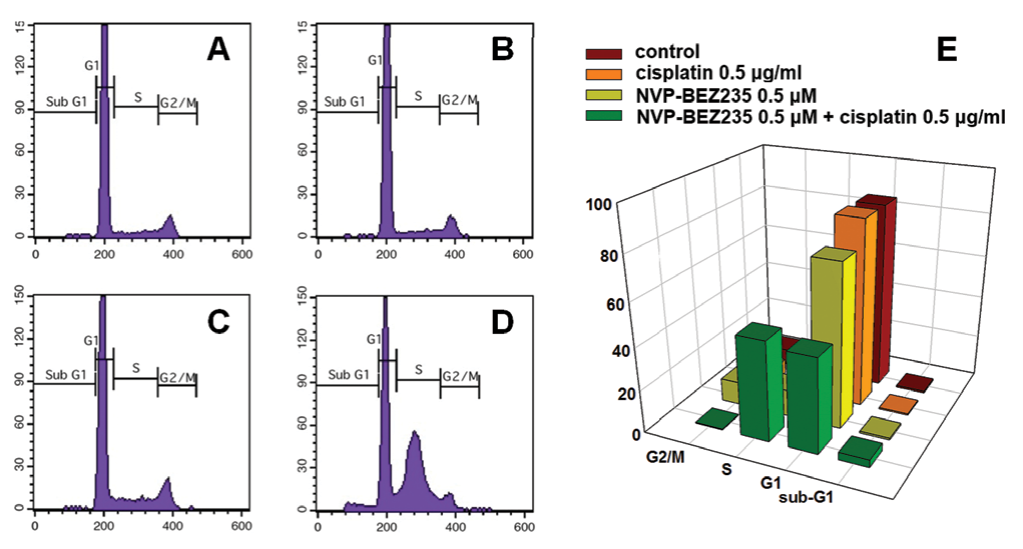
Representative flow cytometric DNA content histogram of untreated control (A) and T24R2 cells exposed to suboptimal dose of cisplatin (B), NVP-BEZ235 (C) alone or in combination (D) for 72 h. The quantitative analysis of the results is shown in (E) where each data point represents the mean ± SD of duplicate experiments.

Neither NVP-BEZ235 (0.5 μM) nor cisplatin (0.5 μg/ml) single-agent treatment caused any significant changes in the cell cycle distribution, while the concomitant treatment resulted in a marked increase of cells in the sub-G1 (3.6±0.6%) and S (46.9±4.3%) phase compared with the untreated control (0.8±0.3% for sub-G1, 5.16±1.1% for S phase), NVP-BEZ235 (0.84±0.3% for sub-G1, 12.6±2.1% for S phase), or cisplatin (0.61±0.3% for sub-G1, 5.49±0.1.3% for S) single-treated cells (Fig. 5).

**Figure 6:**
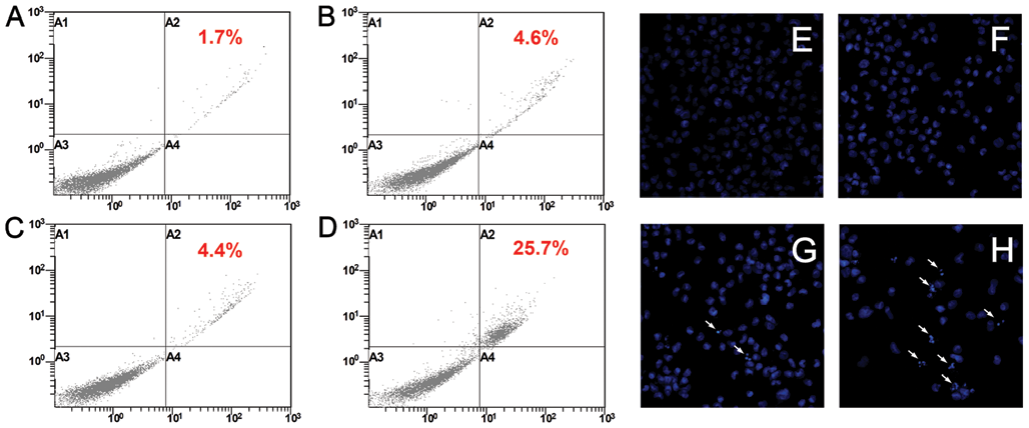
T24R2 cells were treated with cisplatin [(B and F)/0.5 μg/ml], NVP-BEZ235 [(C and G)/0.5 μM)] alone or in combination (D and H) for 72 h and induction of apoptosis was assessed by Annexin V-FITC/PI flow cytometry (left panel) in which A3, Annexin V^−^/PI^−,^ representing viable cells; A4, Annexin V^+^/PI^−,^ representing early apoptotic cells; A2, Annexin V^+^/PI^+^ cells, representing late apoptotic cells; and A1, Annexin V^−^/PI^+^ cells, representing genuine necrotic response, respectively. Hoechst 33342 nuclear stating (x200, right panel). (A and E) untreated controls. Arrows indicate apoptotic bodies formed in T24R2 cells.

Flow cytometric analysis after Annexin V-FITC/PI double stating showed significantly increased population of Annexin V-positive and PI-negative cells (in late apoptotic stage) in concomitant treatment group (25.7±3.6%) compared with untreated control (1.7±0.7%) and cisplatin (4.6±1.3%) or NVP-BEZ235 (4.4±3.6%) single treatment groups (p<0.05, Fig. 6).

Hoechst 33342 nuclear staining showed more frequency chromatin fragmentation and condensation in T24R2 cells exposed to concomitant treatment compared with untreated control and also cisplatin or NVP-BEZ235 single agent-treated groups (Fig. 6).

### Expression of apoptosis, cell cycle and survival regulators

**Figure 7:**
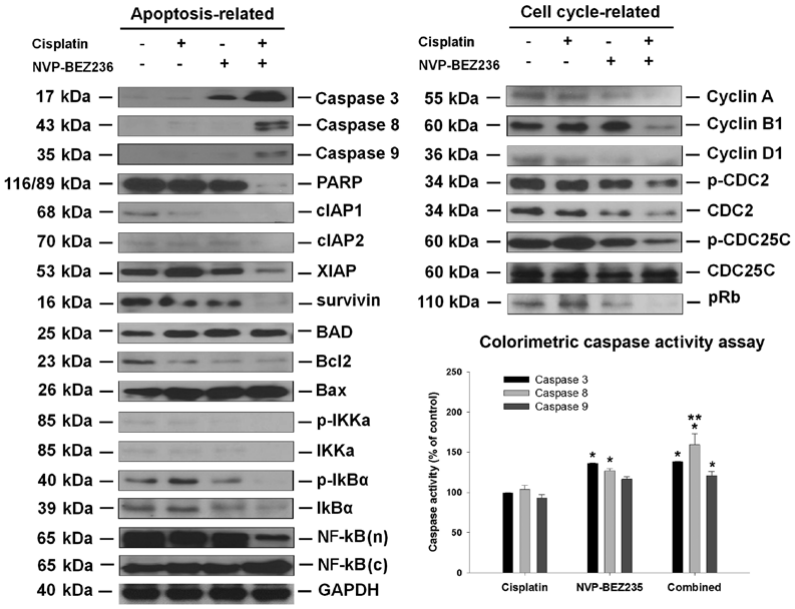
Apoptosis and cell cycle regulatory protein expression in T24R2 cells. T24R2 cells were exposed to NVP-BEZ235 (0.5 μM) and/or cisplatin (0.5 μg/ml) for 72 h and apoptosis- [cleaved caspase (-3, -8 and -9), PARP, cIAP1, cIAP2, XIAP, survivin, BAD, Bcl-2, Bax, p-IKKα, IKKa, p-IκBα, IκBα and NF-κB] and cell cycle-related (cyclin A, cyclin B1, cyclin D1, pCDC2, CDC2, pCDC25C, CDC25C and pRb) protein expression was analyzed by western blot analysis. Caspase activity was also quantitatively analyzed using colorimetric caspase activity assay kit. Asterisks indicate significant difference between each treatment group and untreated control. Double asterisk denotes significant difference between NVP-BEZ235 single and cisplatin/NVP-NEZ235 combined treatment groups.

**Figure 8:**
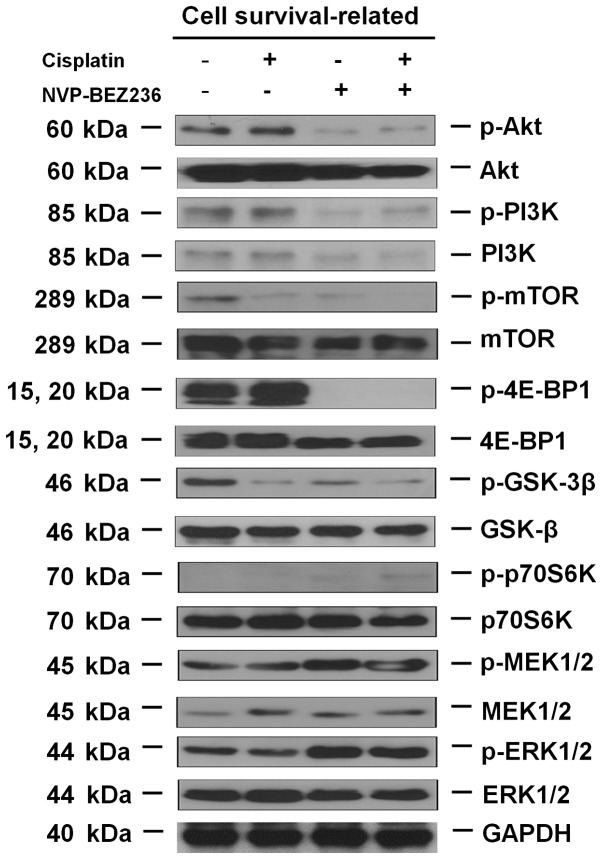
Cell survival-related protein expression in T24R2 cells. T24R2 cells were treated with NVP-BEZ235 (0.5 μM) alone or in combination with cisplatin (0.5 μg/ml) for 72 h and expression of cell survival-related proteins (p-Akt, Akt, p-PI3K, PI3K, p-mTOR, mTOR, p-4E-BP1, 4E-BP1, p-GSK-3β, GSK-3β, p-p70S6K, p70S6K, p-MEK1/2, MEK1/2, p-ERK1/2 and ERK1/2) was analyzed by western blot analysis.

The combined treatment of T24R2 cells with NVP-BEZ235 and cisplatin markedly enhanced the cleavage of caspase-3, -8, and -9 accompanied by increased PARP cleavage (Fig. 7). A colorimetric assay also exhibited increased caspase-3, -8, and -9 activity in concomitant treatment group (169.4±3.8, 149.7±1.2 and 148.7±2.9% of untreated controls, respectively, Fig. 7). Combination treatment suppressed the expression or activation of anti-apoptotic (cIAP2, XIAP and surviving) and cell cycle regulatory protein (cyclin A, cyclin B, pCDC2C, CDC2C, pCDC25C, CDC25C, and pRb expression, Fig. 7). The phosphorylations of cell survival-regulatory proteins (PI3K, Akt, mTOR, 4E-BP1, GSK-3β and p70S6k) were efficiently suppressed by combination treatment (Fig. 8). Both the NVP-BEZ235 single and the combination treatment with cisplatin increased the phosphorylation of MEK1/2 and ERK1/2 in T24R2 cells (Fig. 8).

## Discussion

The sensitivity of cancer cells to chemotherapeutic drug-induced apoptosis depends on the balance between pro-apoptotic and anti-apoptotic signals. Therefore, targeting of anti-apoptotic signals that promote cell survival is proposed as a promising strategy to enhance the efficacy of conventional chemotherapeutic agents. One signaling pathway that has recently drawn much attention for this purpose is the PI3K/AKT/mTOR axis and abnormal activation of this pathway has been reported to play an important role in progression, metastasis, and also chemoresistance in a variety of tumors including bladder cancer (22–26). These findings ignited enthusiasm for targeting mTOR signaling as an anticancer modality and led to the development and clinical application of mTOR inhibitors such as rapamycin, temsirolimus, and everolimus. Although these 1st generation rapalogs have shown promise, due to their allosteric inhibition of mTORC1, but not mTORC2, they usually permit mTORC2-mediated Akt phosphorylation, causing paradoxical activation of survival axis signaling (27–29). Thus, the concomitant dual inhibition of PI3K/mTOR or Akt/mTOR may be a solution to these feedback loops and provide a superior strategy for overcoming development of resistance of cancer cells to targeted therapy.

In the present study we demonstrated dose-dependent antitumor effect of PI3K/mTOR dual inhibitor NVP-BEZ235 in bladder, prostate and kidney cancer cell lines. In bladder cancer cells, NVP-BEZ235 showed relatively more potent antitumor effect than mTOR inhibitor such as temsirolimus and everolimus. In addition, NVP-BEZ235 prevented the negative feedback activation of Akt usually observed after treatment with 1st generation rapalogues. However, relatively high dose of NVP-BEZ235 was required for the growth inhibition of bladder cancer cells and even at the dose of 0.5 μM or higher, NVP-BEZ235 caused only partial inhibition of bladder cancer cells. This is compatible with a previous report in which NVP-BEZ235 showed only partial inhibition (~60%) of prostate cancer cell growth at the concentration of 0.5 μM (30).

Also in bladder cancer cells, NVP-BEZ235 showed only a transient effect resulting in partial loss of its antitumor activity 3 days after initial treatment and this phenomenon was more prominent in cisplatin-resistant T24R2 cells compared with cisplatin-sensitive parental T24 cells. These findings are compatible with previous studies in which mTOR inhibitors showed only cytostatic and transient antitumor effect in many tumors including bladder cancer cells (13,31). Moreover, T24R2 cells showed significantly higher resistance to NVP-BEZ235 compared with other bladder cancer cell lines including cisplatin-sensitive T24. These findings suggest possible cross-resistance between NVP-BEZ235 and cisplatin in human bladder cancers, and thus limited antitumor effect of NVP-BEZ235 in the patients with cisplatin-resistant bladder cancer, a potential target population for these novel therapies.

Based on previous reports that the mTOR inhibitors can exert synergism with various conventional chemotherapeutic agents including cisplatin in chemo-naïve or resistant tumors, we reasoned that the combination of NVP-BEZ235 and cisplatin might enhance antitumor effect of both agents in cisplatin-resistant bladder cancer cells (17,32,33). To test this hypothesis, we performed a synergy test for the NVP-BEZ235 and cisplatin and found a significant enhancement in the antitumor effect compared with that of either agent as a single treatment. The fa-CI plot showed that the two drugs exert synergistic effect over a wide range of dose combinations in cisplatin-resistant T24R2 cells. Dose-reduction index (DRI) analysis demonstrated that when used in combination to treat T24R2 cells, the IC_50_ of cisplatin and NVP-BEZ235 can be reduced by 3.6- and 5.6-fold, respectively, indicating strong synergistic interaction between the two drugs. 3D synergy test resulted in synergy volume 388.25 μM/ml^2^% with minimal antagonism. According to previous guidelines such an extent of the synergy volume indicates that this effect may be important *in vivo* (19).

Flow cytometry showed that combination of NVP-BEZ235 and cisplatin causes a mild increase in sub-G1 fraction while inducing marked increase in S phase population suggesting prominent cytostatic effect of combined treatment rather than apoptogenic effect. Western blot analysis demonstrated that combined treatment caused a marked decrease in cyclin A, cyclin B1, cyclin D1, pCDC2C, CDC2C, pCDC25C, CDC25C, and pRb in T24R2 cells, supporting the flow cytometry data of cell cycle arrest. The combination treatment also caused a significant decrease in the anti-apoptotic cIAP1, cIAP2, XIAP, survivin, and Bcl-2 expression, while causing upregulation of proapoptotic Bad, and Bax expression. Both western blot analysis and colorimetric assay exhibited increased cleavage of caspase-3, -8, and -9 in NVP-BEZ235 and cisplatin co-treated T24R2 cells indicating induction of the caspase-dependent apoptotic pathway. Flow cytometric analysis after Annexin V-FITC/PI double staining and Hoechst 33342 nuclear stating also showed increased apoptosis in concomitant treatment group.

The exposure of T24R2 cells to concomitant treatment suppressed the phosphorylation of IκB kinase α (p-IKKα) and IκBα in conjunction with an increase in cytoplasmic NF-κB and reciprocal decrease of nucleic NF-κB levels, suggesting the suppression of NF-κB signaling by NVP-BEZ235 and cisplatin co-treatment.

The exposure of T24R2 cells to NVP-BEZ235 alone or in combination with cisplatin resulted in the suppression of PI3K and mTOR phosphorylation, as well as its immediate target GSK-3β and 4E-BP1, which was accompanied by slight increased expression or activities of its downstream target BAD, caspase-3 and -9 and accompanying suppression of Bcl-2, cyclin A, and D1. NVP-BEZ235 and/or cisplatin treatment suppressed Akt phosphorylation without any paradoxical activation which was reported in 1st generation mTOR inhibitors such as temsirolimus and everolimus. Akt promotes cell cycle progression through the inhibition of GSK-3β and it suppresses the expression of the Bcl-2 antagonist Bad, maintaining cell survival (34). Thus simultaneous suppression of PI3K and mTOR without activation of Akt and its downstream target signaling also supports the potent antiproliferative and proapoptotic activities of NVP-BEZ235 and cisplatin combination treatment in cisplatin-resistant bladder cancer cells.

Interestingly, we found that both NVP-BEZ235 monotherapy and combination treatment caused increased phosphorylation of MEK1/2, and ERK1/2. This result is compatible with recent reports in which NVP-BEZ235 upregulated ERK phosphorylation in Waldenstrom macroglobulinemia cell line although it appeared to be less significant compared with mTOR inhibitor or PI3K inhibitor (35). It has been reported that activation of MAPK/ERK pathway signaling is supposed to be a resistance mechanism in mTOR inhibitor-based therapy and MAPK/ERK inhibitors can improved of antitumor effect of NVP-BEZ235 and other PI3K inhibitors (16,36–39). Thus these findings suggest that concomitant targeting of MAPK/ERK signaling is a promising strategy to enhance antitumor effect of NVP-BEZ235 in bladder cancer patients.

Although the present study has several limitations such as *in vitro* nature of the design and the small number of cell lines tested, it demonstrated synergistic interaction between cisplatin and NVP-BEZ235 in cisplatin-resistant human bladder cancer cells. While NVP-BEZ235 by itself showed only a limited antitumor effect in bladder cancer cells, it synergistically potentiated cisplatin-mediated apoptosis and cell cycle arrest without any paradoxical activation of Akt in cisplatin-resistant human bladder cancer cells. These findings suggest that dual targeting of PI3K/mTOR combined with cisplatin can be a promising strategy for the patients with cisplatin-resistant bladder cancer. Also our data indicate possible crosstalk between PI3K/Akt/mTOR and MAPK/ERK pathway, thus suggesting the potential of concomitant targeting of MAPK/ERK pathway to enhance antitumor effect of PI3K/mTOR dual inhibitors in cisplatin-resistant bladder cancer. Further comprehensive molecular studies should be performed to test the safety and *in vivo* synergistic antitumor effect of NVP-EBZ235 and cisplatin combination therapy for the clinical application in cisplatin-resistant bladder cancer.

## Figures and Tables

**Table I:** Dose-effect relationship parameters of cisplatin, NVP-BEZ235 and combination treatment of T24R2 cells *in vitro*.

Compound	m^a^	Dm^b^	r^c^
Cisplatin	1.076	23.895	0.941
NVP-BEZ235	9.895	37.476	0.988
Cisplatin + NVP-BVEZ235	1.353	6.629	0.926

a m is a coefficient signifying the shape of the dose-effect curve.

b Dm (IC_50_) is the dose of drugs to produce 50% inhibition of cell proliferation.

c r is the correlation coefficient signifying conformity of the data to the mass action law.

**Table II:** Dose reduction index (DRI) for T24R2 cells.

fa^a^	0.1	0.2	0.3	0.4	0.5	0.6	0.7	0.8	0.9
Cisplatin	2.4	2.8	3.1	3.3	3.6	3.9	4.2	4.7	5.5
NVP-BEZ235	2.5	3.4	4.1	4.9	5.7	6.6	7.8	9.5	13.0

a fa, the fraction affected (e.g., fa 0.5 is equal to a 50% reduction of cell proliferation).
